# Circadian Control of Global Transcription

**DOI:** 10.1155/2015/187809

**Published:** 2015-11-23

**Authors:** Shujing Li, Luoying Zhang

**Affiliations:** ^1^College of Life Science & Technology, Huazhong University of Science and Technology, Wuhan, Hubei 430074, China; ^2^Key Laboratory of Molecular Biophysics of the Ministry of Education, Wuhan, Hubei 430074, China

## Abstract

Circadian rhythms exist in most if not all organisms on the Earth and manifest in various aspects of physiology and behavior. These rhythmic processes are believed to be driven by endogenous molecular clocks that regulate rhythmic expression of clock-controlled genes (CCGs). CCGs consist of a significant portion of the genome and are involved in diverse biological pathways. The transcription of CCGs is tuned by rhythmic actions of transcription factors and circadian alterations in chromatin. Here, we review the circadian control of CCG transcription in five model organisms that are widely used, including cyanobacterium, fungus, plant, fruit fly, and mouse. Comparing the similarity and differences in the five organisms could help us better understand the function of the circadian clock, as well as its output mechanisms adapted to meet the demands of diverse environmental conditions.

## 1. Introduction

Circadian rhythms, controlled by endogenous circadian clocks, are rhythmic oscillations in our behavior and physiological processes with a period close to 24 h. Circadian rhythm exists in diverse organisms on the Earth ranging from bacteria and fungi to plants and animals, allowing adaptation to light and temperature changes caused by the self-rotation of the Earth [1, 2]. In all kingdoms of life, the circadian clock regulates a wide variety of physiological activities such as cyanobacteria cell division [3], fungal sporulation [2], plant growth and flowering time [4, 5], and sleep/wake cycles in animals [6].

The circadian clocks are organized around three major physiological components: an input pathway that receives environmental cues and entrain the oscillator, a central oscillator that keeps circadian time and generates rhythms, and an output pathway that generates manifested rhythmic processes throughout the body [7]. The central oscillator in eukaryotic organisms is similar in different kinds of organisms, consisting of transcriptional and posttranscriptional negative feedback loops. In fungi, fruit flies, and mammals, the positive elements of the circadian negative feedback loops are heterodimeric complexes of two PER-ARNT-SIM domain-containing transcription factors that activate the transcription of negative elements. Moreover, the negative elements repress their own expression by inhibiting the activity of the positive elements. The cyclic activation, repression, and reactivation of circadian negative elements generate circadian rhythmicity, which regulates the circadian output pathway by driving downstream clock-controlled gene (CCG) expression [8–10].

## 2. The Core Molecular Clock

Circadian clocks in diverse organisms are composed of molecular feedback loops, but these are coordinated in somewhat different ways with different factors that are presented in detail here, based on our knowledge from five widely used model systems: the freshwater cyanobacterium* Synechococcus elongatus*, the filamentous fungus* Neurospora crassa*, the thale cress* Arabidopsis thaliana*, the fruit fly* Drosophila melanogaster*, and the house mouse* Mus musculus*.

The cyanobacterial clock is regulated by the activity of three genes,* kaiA*,* kaiB*, and* kaiC*. Inactivation of any of the* kai* genes abolishes clock function [11]. The phosphorylation status of KaiC exhibits robust circadian oscillation [12]. KaiC also exhibits ATPase activity, which correlates with clock speed [13]. Kai proteins interact with each other and regulate the rhythmic supercoiling/condensation status of the chromosome [14, 15]. This supercoiling/condensation status of the chromosome rhythmically changes such that it becomes an oscillating nucleoid, or oscilloid, which globally regulates rhythmic gene expression [16].

In the core* Neurospora* circadian clock, the positive element is the heterodimeric White Collar Complex (WCC) consisting of WC-1 and WC-2, and the key negative element is the FREQUENCY- (FRQ-) FRQ RNA helicase (FRH) complex [17–19]. WCC binds to* frq* promoter and activates* frq* transcription. Meanwhile, the FRQ-FRH complex (FFC) recruits the casein kinases to phosphorylate WC proteins which lead to dissociation of WCC from the* frq* promoter, thereby inhibiting transcription of* frq* and closing the negative feedback loop [20–23]. FRQ undergoes progressive phosphorylation by several kinases and is degraded through the ubiquitin proteasome pathway [20, 24]. After the degradation of FRQ protein, WCC reactivates* frq* transcription, thereby initiating a new circadian transcriptional cycle. The cyclic activation, repression, and reactivation of* frq* expression bring about circadian oscillation, which is the major basis of the rhythmic expression of CCGs. In addition to the role in repressing WCC function in the negative feedback loop, FRQ functions to promote the steady-state levels of WC-1 and WC-2, forming a positive feedback loop [25]. These interconnected feedback loops are essential to maintain the robust and stable oscillation in* Neurospora*.

In* Arabidopsis*, the central oscillator consists of three interlocked transcriptional feedback loops, the core loop, the morning loop, and the evening loop. The core loop consists of two single MYB transcription factors, Circadian Clock Associated 1 (CCA1) and Late Elongated Hypocotyl (LHY), which repress the expression of evening-phased pseudoresponse regulator (*PRR*) and* Timing of CAB Expression 1* (*TOC1*) [26–30]. TOC1 originally was reported to activate* CCA1* and* LHY* expression [26], but more recent reports have confirmed that TOC1 also inhibits the expression of* CCA1* and* LHY* [31, 32]. Members of the PRR family (PRR5, PRR7, and PRR9) bind to promoters of their activators,* CCA1* and* LHY*, and repress their expression, forming a second interlocked morning loop [33, 34]. The evening loop is composed of TOC1, GIGANTEA (GI), and the evening complex (EC) including Early Flowering 3 (ELF3), ELF4, and LUX ARRYTHMO (LUX)/PHYTOCLOCK 1 acting at dusk as a transcriptional repressor of* PRR9* expression [35–37].

The* Drosophila* circadian oscillator is composed of two interlocked feedback loops. In the core feedback loop,* period* (*per*) and* timeless* (*tim*) transcription are activated when CLOCK (CLK) and its heterodimeric partner CYCLE (CYC) bind E-box elements in* per* and* tim* promoters [38–41]. As* per* mRNA accumulates to peak levels around dusk, PER accumulates in the cytoplasm, where it binds TIM and then translocates into the nucleus, thereby inhibiting CLK/CYC activity and subsequently repressing the transcription of* per* and* tim* [42–44]. Once TIM is induced to degrade early in the light phase, thus “deprotecting” PER which is also targeted for degradation, CLK/CYC binds to E-boxes again to initiate the next cycle of* per* and* tim* transcription [45–47]. In the second feedback loop, CLK/CYC drives the transcription of* vrille* (*vri*) and* PAR-domain protein 1ε* (*Pdp1ε*), whose protein products repress and activate the transcription of* clk*, respectively [48, 49].

The circadian oscillator in mouse is built on a series of feedback loops highly similar to that in* Drosophila*. The core feedback loop contains a heterodimer of transcriptional activators formed by brain and muscle ARNTL-like protein 1 (BMAL1) and circadian locomotor output cycles kaput (CLOCK), which directs transcription of three Period genes (*Per1*,* Per2,* and* Per3*) and two Cryptochrome genes (*Cry1* and* Cry2*) by binding to E-box sites within their promoters [50–53]. PER and CRY translocate into the nucleus and inhibit the transcriptional activity of CLOCK/BMAL1 [54–56]. Targeted degradation of PER and CRY proteins enables the reactivation of CLOCK/BMAL1, and a new cycle begins [54, 57, 58]. In an additional coupled feedback loop, CLOCK/BMAL1 activates the transcription of retinoic acid-related orphan receptors, Ror*α* and Rev-erb*α*, which activates and represses transcription of* Bmal1*, respectively [53, 59]. In certain tissues, neuronal PAS domain protein 2 (NPAS2) functions as a CLOCK analog [60].

## 3. A Significant Portion of the Transcriptome Is CCGs

As previously mentioned, many genes are rhythmically expressed. In cyanobacteria* Synechococcus elongatus* PCC7942, about 30% to 64% of the transcriptome is expressed in a circadian manner based on results from microarray studies [61, 62]. Circadian genes peak mostly at dawn and dusk, with ~30% more genes peaking at dawn than dusk. Genes that belong to the central intermediary metabolism, including glycoprotein and polysaccharide synthesis, transcription, and energy metabolism, are enriched among the rhythmically expressed transcripts [61].

In* Neurospora*, high-density microarrays demonstrated that roughly 20% to 25% of the transcriptome can be expressed under circadian control [63, 64]. Very recently, RNA sequencing (RNA-Seq) revealed that from 10% to as much as 40% of the transcriptome is under the control of the clock [65, 66]. Oscillating genes are enriched in pathways involving metabolism, protein synthesis, stress responses, cell signaling, and development [63–66]. Similar to cyanobacteria, the peak time of* Neurospora* CCG expression is also clustered in either dawn or dusk [65, 66]. In general, dawn-phased genes are mainly participating in catabolic processes of energy production and precursor assembly, whereas dusk-phased genes are mostly involved in anabolic processes of cellular components and growth.

In* Arabidopsis*, between 6% and 15% of the transcriptome is regulated by the circadian clock [67–69], and by combining the three data sets and thus improving the strength of the analysis, between 31% and 41% of the expressed genes are believed to oscillate [70]. This is consistent with an enhancer trap study showing that roughly one-third of the genome is rhythmically regulated [71]. Another study investigated the transcriptome under different thermocycles, photocycles, and circadian conditions and found that 89% of the transcripts oscillate in at least one of the conditions [72]. CCGs are overrepresented among all of the classical plant hormone and multiple stress response pathways, as well as cell cycle and protein synthesis [70, 72].

Based on microarray studies, approximately 1% of genes from* Drosophila* head exhibit circadian expression pattern [73–77]. Recently, RNA-Seq assays revealed that close to 2% of the genes in the fly head and 4% in the fly brain demonstrate rhythmic expression, including several noncoding RNAs that were not identified in microarray studies [78, 79]. To distinguish transcriptional versus posttranscriptional regulations in the transcriptome, nascent RNAs from fly heads were isolated and subjected to high-throughput sequencing (Nascent-Seq) [79]. 130 robust cycling transcriptional units were detected, which is about 1% of the genome, and more than 1/3 of these transcripts exhibit oscillation in mRNA analysis. The reverse comparison indicates that 19% of the cycling mRNAs are identified to be cycling in the Nascent-Seq data, implicating significant contribution of posttranscriptional modifications that contribute to rhythmic expression of CCGs [79]. CCGs in fly heads are associated with diverse biological processes, including areas of metabolism, detoxification, signal transduction, and immunity [73–77, 79].

In mouse, approximately 5% to 25% of the expressed genes in central and peripheral tissues were identified as oscillating according to microarray and RNA-Seq studies [80–84]. Despite being greatly informative, most of these studies have analyzed only one or two organs/tissues. A recent study, which profiled the transcriptomes of 12 different mouse organs, reported that 43% of all protein coding genes exhibit circadian rhythms of mRNA abundance somewhere in the body, largely in an organ-specific manner, and 32% of conserved noncoding RNAs oscillate as well [85]. Consistent with the findings in* Drosophila*, Nascent-Seq demonstrated that roughly 15% of all detected genes are rhythmically transcribed in the mouse liver, but of which only 42% exhibit mRNA oscillations [86]. On the other hand, about 70% of the genes that show rhythmic mRNA expression do not show transcriptional rhythms, indicating the existence of substantial posttranscriptional regulation that leads to mRNA cycling. Mouse CCGs are involved in diverse biological pathways, particularly various metabolic pathways, along with many others [81–83, 86].

## 4. Transcriptional Regulation of CCGs

How are these CCGs rhythmically transcribed? Based on our current understanding, this is accomplished by coordinated efforts of rhythmic activities of transcription factors at promoter elements in the genome and rhythmic epigenetic modifications, such as chromatin remodeling through posttranslational modifications (PTMs). This will be described in detail below.

In cyanobacteria, the KaiC-containing protein complex regulates circadian gene expression via multiple protein-dependent pathways [87]. In one pathway, KaiC interacts with a histidine kinase SasA, which contains a KaiB-like sensory domain [88]. KaiC increases the rate at which SasA autophosphorylates, and the autokinase activity of SasA is crucial to its function [88–90]. SasA phosphorylates and activates a transcription factor RpaA, which regulates the expression of a small set of circadian effectors that orchestrate genome-wide transcriptional rhythms [87, 89, 91]. More specifically, RpaA functions to promote dusk-like expression state [89]. In parallel to SasA, low amplitude and bright (LabA) is also believed to signal to RpaA and represses circadian gene expression [92]. A third pathway involving CikA exerts repressive effects on circadian gene expression, possibly by promoting dephosphorylation and suppressing RpaA activity [89, 92]. The phosphatase activity of CikA is enhanced by KaiB/C at a time that is distinct from the activation of SasA by KaiC [89]. The RpaA paralog, RpaB, is recently shown to bind rhythmically to several promoters, including* kaiBC* promoter in the subjective night, and repress transcription [92]. This binding may be terminated by RpaA to activate transcription during the subjective day. Moreover, the core clock proteins KaiA and KaiC exert opposite effects on global circadian gene expression [93]. KaiA overexpression activates “dusk genes” and represses “dawn genes,” whereas KaiC overexpression results in the opposite effect, that is, repression of “dusk genes” and activation of “dawn genes.” In addition to protein-dependent pathways, kai proteins regulate chromosome compaction rhythm and oscillation in the superhelical status of the DNA [90, 94]. The topological state of the chromosome is highly correlated with gene expression, and depending on the AT content in the promoter regions, some genes are activated while some genes are repressed by chromosome relaxation [62, 95]. An oscilloid model proposes that topological changes of the chromosome mediated by KaiABC oscillator drive cyclic expression of genes at a global scale, and specific promoter elements are believed to be not essential [96].

In* Neurospora*, microarray and RNA sequencing studies demonstrate that 10–40% of the genome is circadianly expressed, and these genes contain WCC binding sites and light response elements (LRE), as well as a number of other motifs in their promoter regions [63–65, 97]. Chromatin immunoprecipitation (ChIP) results show that WC-2 is physically associated with 300 to over 400 regions in the genome, depending on the environmental conditions [65, 98]. Interestingly, the expression of 8–20% of transcription factor genes is regulated by WC-2 [65, 98, 99]. The authors propose that these transcription factors regulated by WCC can control downstream target genes, thus establishing a hierarchical system that shapes genome-wide rhythmic expression of CCGs. One such transcription factor driven by WCC is CSP1, a transcriptional repressor [100]. Genes controlled by CSP1 are rhythmically expressed with a peak in the evening and are predominantly involved in anabolic processes, whereas genes strongly dependent on WCC peak in the morning and are mainly involved in catabolic processes [97, 100]. The* Neurospora* clock has also been shown to regulate the phosphorylation and thus activation of mitogen-activated protein kinases MAK-1 and MAK-2 [101]. MAK-1 is required for normal expression of at least 145 CCGs. Moreover, RNA polymerase II (Pol II) is rhythmically recruited to over 1300 genes and about 25% of these genes display rhythms in transcript levels [97].

In* Arabidopsis*, promoter analyses of genes with cycling mRNA levels, in combination with luciferase reporter and enhancer trap assays, identified the evening element (EE), which is overrepresented in the promoters of evening-phased genes and both necessary and sufficient for transcription occurring in the evening [69, 71, 102, 103]. CCA1-binding site (CBS), which is only one base pair different from the EE, is important for morning-phased transcription [71, 103]. Two additional* cis*-regulatory elements, the morning element and protein box, mediate transcription in the morning and midnight, respectively [102, 104]. Recently, using ChIP followed by deep sequencing, over 500 genomic regions were found to be targeted by PRR5 and over 1000 regions by CCA1 [105, 106]. PRR5 direct targets are enriched in transcription factors, providing a means for PRR5 to control clock output, that is, CCGs [106]. PRR5 represses the expression of its direct targets from noon to midnight, possibly in conjunction with PRR7 and PRR9. The majority of the genes associated with strongest CCA1-binding peaks contain EE and show a rhythmic pattern with the peak of expression in the evening [105].

In* Drosophila*, a study using ChIP tiling array assays has demonstrated that CLK binds to ~1500 sites in the genome, and association with at least ~60% of these sites appears to be rhythmic [107]. These target regions are enriched for canonical or degenerate E-boxes. CYC is detected and PER binds to CLK/CYC about 4–6 h later at most of these target sites, which suggests that these target genes are regulated similarly to core clock genes. Pol II binds rhythmically to approximately ~30% of the target genes, leading to cyclic synthesis of RNA [107].

In the mouse, most of the core clock proteins, including BMAL1, CLOCK, NPAS2, PER1, PER2, CRY1, CRY2, and REV-ERBs, have all been shown to bind to over 1000 sites in the genome with a circadian rhythm [81, 108, 109]. These target sites are enriched in E-boxes, as well as binding motifs for CEBPA and a number of nuclear receptors. Pol II is cyclically recruited to the genome with a peak that coincides with the peak of global rhythms in nascent transcription [81]. Genome-wide analysis revealed that the majority of the expressed genes undergo circadian histone modifications regardless of whether RNA oscillation can be detected and the recruitment (and initiation) of Pol II may contribute to variation in the amplitude of histone marks [81, 84, 110]. Valekunja and colleagues identified the histone-remodeling enzyme mixed-lineage leukemia 3 (MLL3) as a CCG that can modulate over 100 epigenetically targeted circadian output genes. Moreover, inactivating the methyltransferase activity of MLL3 severely impairs cyclic methylation at the promoters of core clock genes [111]. Genome-wide histone acetylation also exhibits a diurnal rhythm, contributing to rhythmic expression of CCGs [112]. This is believed to be orchestrated at least in part by histone deacetylase 3 (HDAC3), which is rhythmically recruited to the genome by REV-ERB*α*. Another study employed chromosome conformation capture on chip technology and demonstrated oscillation in spatial and temporal chromosomal organization, which is driven by the clock [113]. This may lead to rhythmic generation of genomic environments that promote rhythmic gene expression. Recently, enhancer RNAs have also been identified which modulate genome-wide rhythmic expression of CCGs [114].

## 5. Conclusion and Discussion

CCGs consist of a substantial portion of the genome. Although they participate in diverse biological processes that differ in different organisms and tissues/organs, genes involved in metabolism appear to be circadianly regulated throughout the phylogeny. This implicates an evolutionarily conserved function of the circadian clock in regulating rhythmic metabolic processes. This also means that it is particularly important for metabolic processes to be synchronized to the external day/night cycle.

The transcription of CCGs is regulated by rhythmic activities of transcription factors accompanied by rhythmic alterations of the chromatin in organisms ranging from cyanobacteria to plants and mammals, reflecting evolutionarily conserved mechanisms controlling circadian gene transcription. However, the complexity of the regulations increased by orders of magnitude. In the unicellular prokaryote cyanobacteria, CCGs are regulated by a few transcription factors as well as rhythmic changes in the topology of the chromosome, and no specific promoter sequences are believed to be involved. Apparently, this is not sufficient for more complex organisms to adapt to the daily cycle, and thus specific* cis*-elements evolved, along with many more transcription factors and various posttranslational modifications of the chromatin to modulate rhythmic expression of CCGs. The combined actions of these processes result in unique circadian transcriptomes adapted to the needs of each organism, as well as each tissue/organ within an organism. It would be of interest to identify common and distinct mechanisms employed by diverse organisms and tissues/organs. The former shall reveal fundamental pathways adopted by the clock output system, whereas the latter will reflect unique output mechanisms that are a result of unique clock-environment interactions.
