# Corrigendum to “Circadian Control of Global Transcription”

**DOI:** 10.1155/2016/8369785

**Published:** 2016-07-19

**Authors:** Shujing Li, Luoying Zhang

**Affiliations:** ^1^College of Life Science & Technology, Huazhong University of Science and Technology, Wuhan, Hubei 430074, China; ^2^Key Laboratory of Molecular Biophysics of the Ministry of Education, Wuhan, Hubei 430074, China; ^3^Department of Life Sciences, Bengbu Medical College, Bengbu, Anhui 233030, China

In the article titled “Circadian Control of Global Transcription” [1], there was a missing affiliation for the first author. The corrected author list and affiliation are shown above.
